# Training and experience outperform literacy and formal education as predictors of community health worker knowledge and performance, results from Rongo sub-county, Kenya

**DOI:** 10.3389/fpubh.2023.1120922

**Published:** 2023-04-27

**Authors:** Ash Rogers, Lou L. Goore, Jane Wamae, Joseph R. Starnes, Stephen O Okong’o, Vincent Okoth, Sandra Mudhune

**Affiliations:** ^1^Lwala Community Alliance, Rongo, Kenya; ^2^Department of Pediatrics, Division of Pediatric Cardiology, Vanderbilt University Medical Center, Nashville, TN, United States

**Keywords:** community health worker (CHW), community health, Kenya, obstetric danger signs, neonatal danger signs, traditional birth attendant (TBA), resource limited setting

## Abstract

**Introduction:**

There is a growing recognition that Community Health Workers are effective at improving health outcomes and expanding health access. However, the design elements that lead to high-quality Community Health Worker programing are relatively understudied. We looked at the predictors of Community Health Worker knowledge of obstetric and early infant danger signs as well as performance in achieving antenatal care and immunization uptake among their clients.

**Methods:**

The study takes place in the context of an intervention implemented jointly by Lwala Community Alliance and the Kenya Ministry of Health which sought to professionalize Community Health Worker cadres through enhanced training, payment, and supervision. There were four cohorts included in the study. Two cohorts started receiving the intervention prior to the baseline, one cohort received the intervention between the baseline and endline, and a final cohort did not receive the intervention. Data on Community Health Worker demographics, knowledge tests, and key performance indicators were collected for 234 Community Health Workers. Regression analyses were used to explore education, literacy, experience, training, and gender as potential predictors of CHW performance.

**Results:**

We found that clients of Community Health Workers trained through the intervention were 15% more likely to be fully immunized and 14% more likely to have completed four or more antenatal care visits. Additionally, recency of training and experience caring for pregnant women were associated with increased Community Health Worker knowledge. Finally, we found no association between gender and CHW competency and tenuous associations between education/literacy and Community Health Worker competency.

**Discussion:**

We conclude that the intervention was predictive of increased Community Health Worker performance and that recency of training and experience were predictive of increased knowledge. Though education and literacy are often used in the selection processes of Community Health Workers globally, the link between these characteristics and Community Health Worker knowledge and performance are mixed. Thus, we encourage further research into the predictive value of common Community Health Worker screening and selection tools. Further, we encourage policymakers and practitioners to reconsider the use of education and literacy as means of Community Health Worker selection.

## 1. Introduction

There has been significant progress in the reduction of maternal deaths in Africa. The maternal mortality ratio dropped from 718 maternal deaths per 100,000 in the year 2000 to 417 maternal deaths per 100,000 live births in the year 2017, with the East African region seeing a decrease from 853 to 443 maternal deaths per 100,000 births (1). This reduction in maternal mortality ratio in Africa represents an annual percentage change of −3.0%. In order to reach the United Nations Sustainable Development Goal of less than 70 maternal deaths per 100,000 live births by 2030 (2), this rate would have to be −15.29%. Further, while Kenya has seen a reduction in maternal mortality on par with the region, those gains are not evenly distributed. Just 15 counties (out of 47) contribute to 98% of all maternal deaths in Kenya (3). Further, the neonatal period is significant for child survival with the risk of death during this period being higher than any other period of childhood. The neonatal mortality rate in Kenya is 1.4 times higher than the postneonatal mortality rate and stands at 22 deaths per 1,000 live births. Additionally, neonatal mortality in Kenya declined at the slowest rate compared to all other childhood mortality between 2003 and 2014 (4).

Community Health Workers (CHWs) can play a pivotal role in increasing health access and tackling maternal and neonatal mortality. Substantial evidence shows the potential of CHWs to increase access to health care, maintain continuity of care during pandemic periods, and ultimately improve health outcomes (5–8).

The Kenyan Government has signaled its commitment to a strong CHW system through Kenya’s Community Health Strategy 2020–2025, which outlines goals for greater CHW coverage, compensation, training, and supervision (9). Migori County, Kenya, the host of this study, passed a new Community Health Services Act in mid-2022, committing the county to greater investment in community health and laying out key commitments to the CHW workforce.

In order for community health programs to deliver on the goals of policymakers, they must be well-designed. The World Health Organization (WHO) Guideline on Health Policy and System Support to Optimize Community Health Worker Programmes provides important guidance on CHW system design elements including recommendations on payment, supportive supervision, and continuous training (10, 11). This guidance is supported by evidence, including a systematic review showing that these design elements are associated with increased performance (12).

However, in these guidelines the WHO highlights gaps in the “evidentiary certainty” of which CHW selection practices lead to quality care (10). Specifically, the guidelines recommend a “minimum education level that is appropriate to the task(s) under consideration” alongside community acceptance, gender equity, and personal attributes like interpersonal skills, life experience and values. The guideline notes that the certainty of evidence for the recommendation is “very low” and considers the recommendation “conditional.” Indeed, research on selection and predictors of CHW performance are limited (13–15).

With this limited direction, selection criteria for CHWs varies widely in the sector, ranging from literacy tests to formal education requirements (16–21). Selection processes are important because they can influence the retention and performance of the community health workforce (22). Additionally, selection can determine the gender balance of these cohorts. Education and literacy tests, for example, risk biasing against women, older populations and other marginalized groups that may be less likely to access formal education (23). Since gender equity is a priority in the best practices laid out by WHO and foundational documents like the CHW AIM Tool, it is important to interrogate practices that may leave women out (11).

Research on the correlation between educational attainment and CHW performance have disparate results with some studies finding associations with education and performance (24–26) and others (27–30) finding no association. Similarly, the literature finds mixed results on the role of gender and years of experience in predicting CHW performance (31). One aspect of CHW selection around which there is wide consensus is the role of community selection. Though the specific methods for community selection lack consistent definition, this general approach is championed by global norm setters and supported in the wider literature (10, 22, 29, 32).

## 2. Methods

### 2.1. Study setting

This study takes place in the context of a professionalized CHW program implemented by the Migori County, Kenya Ministry of Health in partnership with Lwala Community Alliance (Lwala). The intervention works with existing cadres of government CHWs and incorporates practicing traditional birth attendants (TBAs) to create a new cadre of professionalized CHWs, who are then trained, paid, supervised, and equipped to complete their work.

**Figure 1:**
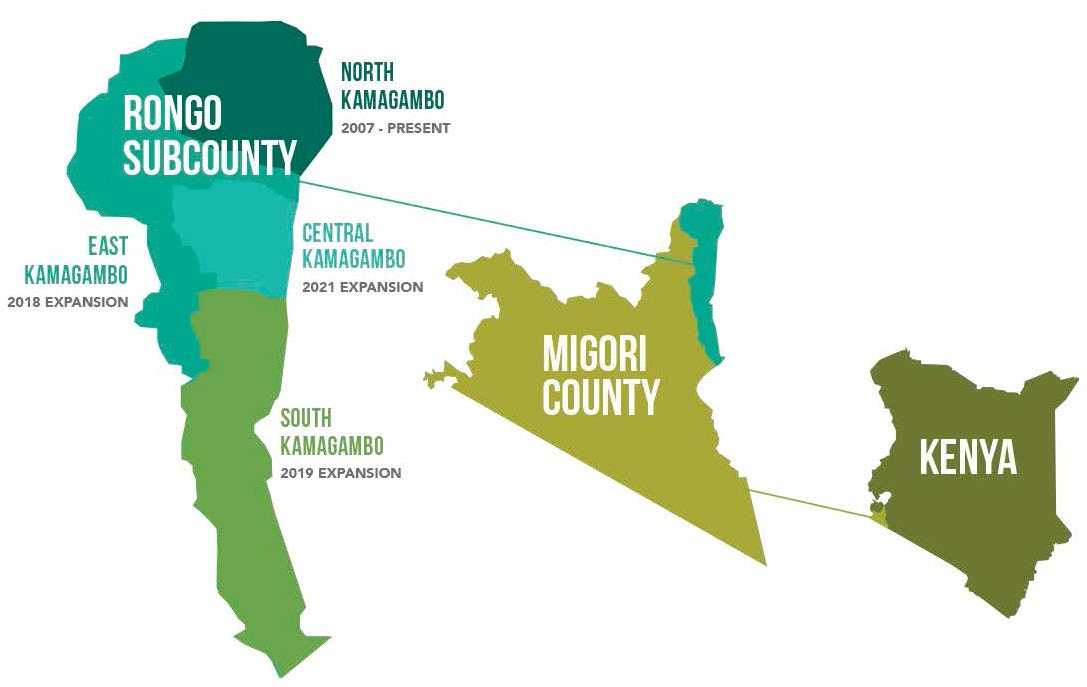
Rongosub-county is located in the northeastern portion of Migori County, Kenya. It is further divided into four wards, which include North Kamagambo and East Kamagambo whereprogramming took place prior to the study, South Kamagambo where programming commenced following the baseline study andCentral Kamagambo, wheretraining had not yet occurred at the end of the study period.

**Figure 2:**
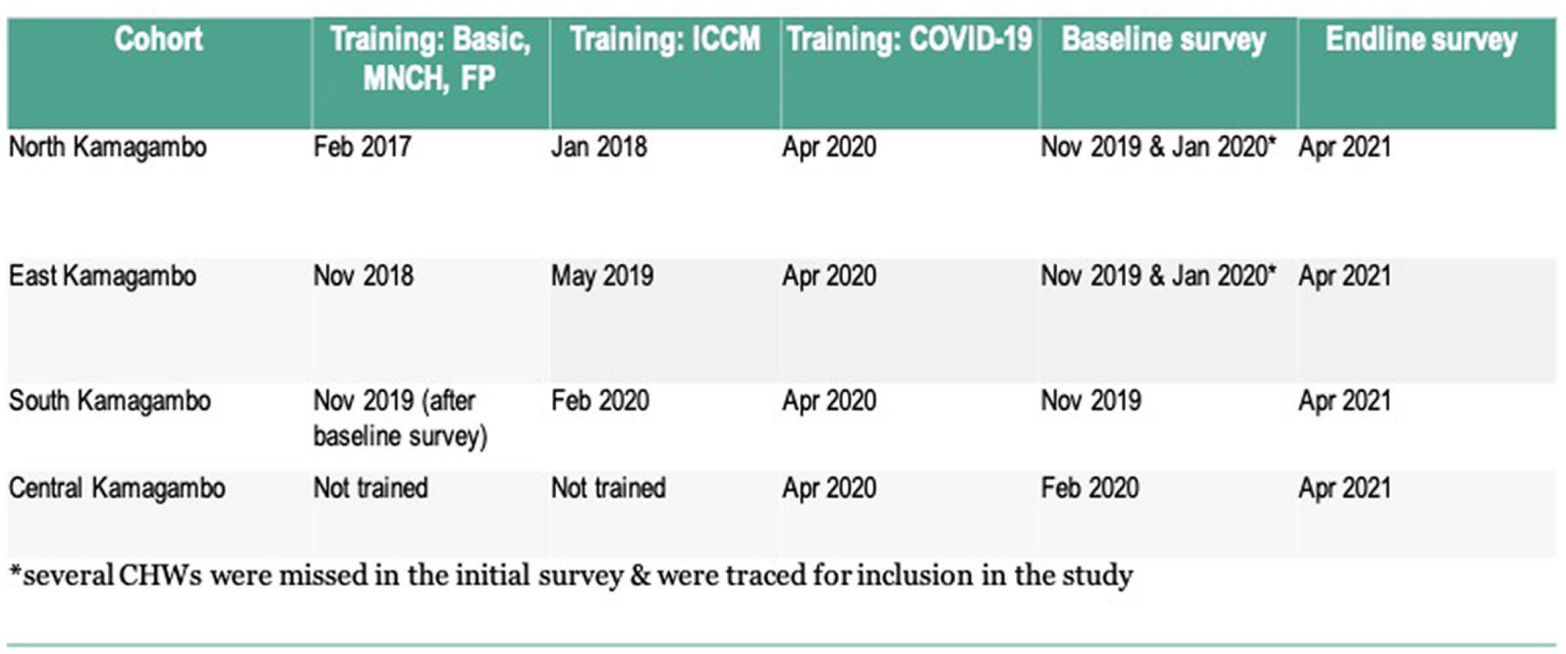
Study timeline. ^*^Several CHWs were missed in the initial survey & were traced for inclusion in the study.

This study explores education, literacy, experience, training, and gender as potential predictors of CHW performance. Data were collected in the Rongo sub-county in the northeastern portion of Migori County, Kenya. Rongo sub-county is further divided into wards, which include North Kamagambo (North), East Kamagambo (East), South Kamagambo (South), and Central Kamagambo (Central). Lwala began working in 2007 in North. Since that time, programming has expanded to all of Rongo sub-county (East Kamagambo, South Kamagambo, and Central Kamagambo). For the purpose of this study, implementation took place in North and East prior to the study period. CHW training and program commencement in South took place immediately following the baseline survey, and training in Central had not yet occurred at the end of the study period (Figures 1, 2).

### 2.2. Intervention

#### 2.2.1. Selection

**Figure 3:**
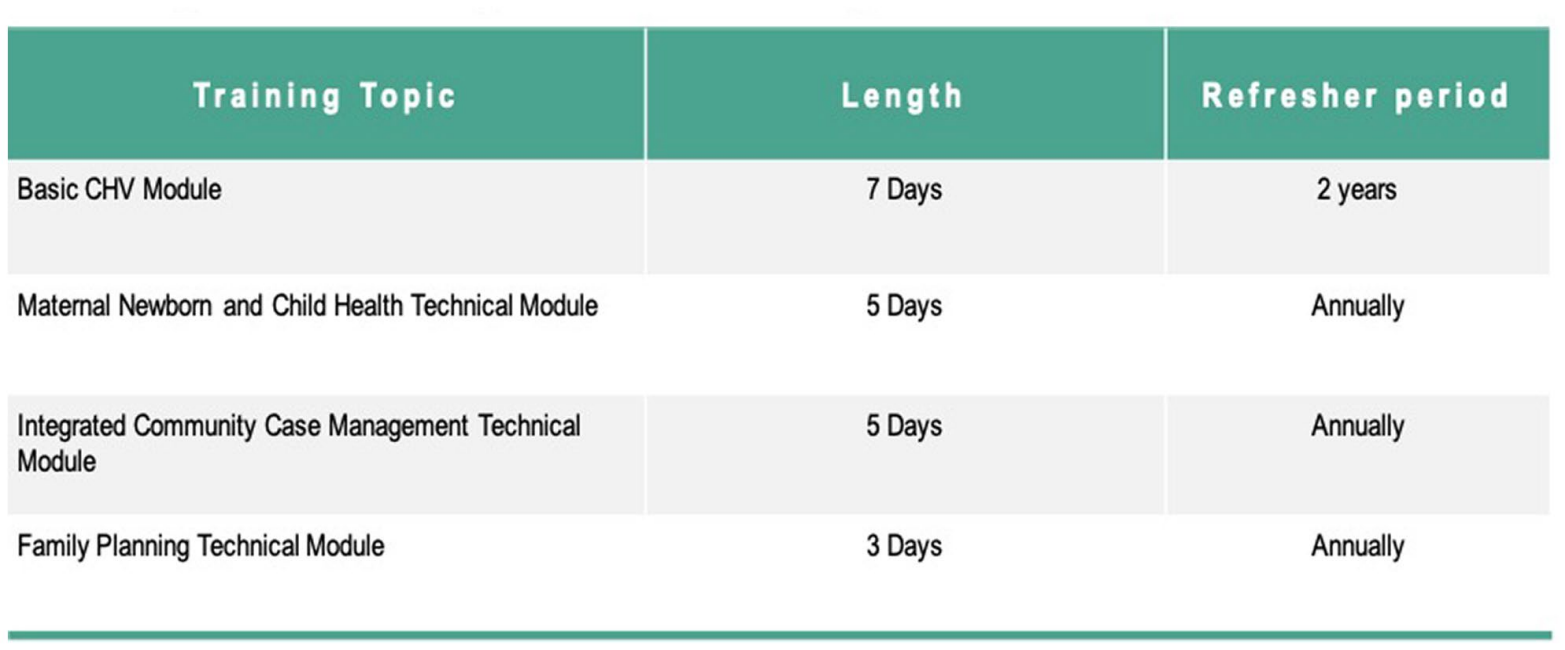
Training topics. All training was based on Kenya’s national curriculum for CHWs.

The existing cadres of government CHWs were originally selected through a participatory process where community members are proposed and endorsed to become CHWs. During the Lwala community entry process in the intervention sites, we selected and worked with all the existing government CHWs and supported a community review process. In addition, TBAs were selected from the community through a community-led mapping process followed by a verification exercise that involved visiting TBAs in their homes. There were no education or literacy requirements imposed, although CHWs in the intervention were required to complete the mandatory training and demonstrate proficiency in the training objectives through written or oral tests (Figure 3). If a CHW did not meet the minimum score they were linked with a strong CHW for mentorship and subjected to another test later.

#### 2.2.2. Training

The intervention included an initial 7 day training on Kenya’s Community Health Volunteer Basic Module, followed by a 5 day training on the Maternal, Newborn, and Child Health Technical Module. As CHWs progressed through the program, they received additional training on technical modules and periodic refresher training as outlined in Figures 2, 3. All training was based on Ministry of Health curriculum and delivered jointly by Migori County Ministry of Health and Lwala. CHWs outside the intervention did not receive training during the study period, with the important exception of training on COVID-19 protocols in April 2020, delivered by Migori County Ministry of Health and Lwala across all cohorts.

#### 2.2.3. Supervision

CHWs in both the intervention and control were supervised by government Community Health Assistants (CHAs). CHAs in the intervention group were trained in the 360 supervision approach. In this approach CHWs participated in weekly review meetings at the village level and monthly review meetings with the link government facility. CHWs also received one-on-one supportive supervision which included CHAs accompanying CHWs on household visits to observe work performance, spot checks of randomly selected households to verify CHW services, and one-on-one meetings with the supervisor to discuss challenges and receive feedback. CHAs outside the intervention did not receive any specific training around CHW supervision, but instead were trained intermittently by government and NGOs on specific public health campaigns.

#### 2.2.4. Payment

CHWs in the intervention received a monthly payment of between 40–60 USD based on the service package they were delivering. CHWs outside the intervention did not receive payment during the study period.

#### 2.2.5. Role

CHWs across the study were responsible for between 60–80 households. CHWs in the intervention were expected to proactively enroll all households in the community and provide preventative and curative services as well as refer clients to government health facilities for care. Core to their responsibilities was providing support to pregnant women to complete antenatal care visits and delivery with a skilled provider as well as track the health of infants and children ensuring early diagnosis and treatment of illness as well as on-time immunizations.

### 2.3. Study design

CHWs were surveyed both at the beginning and end of the study period. The sample size was pre-determined based on the number of CHWs working in the geographic area. Previous studies showed rates of knowledge of neonatal danger signs of 50% among control CHWs and approximately 80% among intervention CHWs (33). Using the chi-squared test, power of 0.8, and alpha of 0.05 gives a sample of just 39 in each group needed to detect a similarly sized difference. We estimated there would be 80–100 CHWs working in each ward, which would give a sample that would provide more than adequate power for the primary outcome of CHW knowledge. The entire CHW population was surveyed both to avoid sampling bias and to provide adequate power for secondary outcomes.

The survey tool was created using validated tools. Knowledge questions were derived from Jhpiego’ training materials (34) and were used in a previous iteration of this survey (33) Perception of supervision was measured using the Perceived Supervision Scale (PSS), which has been validated specifically with CHWs in multiple countries including Kenya (35). Finally, literacy was assessed using a brief assessment based on Kenya’s nationwide literacy exam.

### 2.4. Data collection and management

Surveys were administered by enumerators trained in informed consent, confidentiality, and appropriate survey administration. In order to maximize response rate, surveys were conducted during regularly scheduled CHW trainings and meetings held for each ward. Mobile tablets were used for data collection, which were synced to a central database. Data were routinely checked for internal consistency and outliers.

Data regarding completion of antenatal care visits and immunizations among patients were obtained from de-identified data sources. For CHWs currently employed by Lwala, these metrics are routinely tracked using a tablet-based tool used by CHWs in the field. For those not currently employed by Lwala, these data were obtained from ledgers kept by facilities with which the CHWs were affiliated.

### 2.5. Statistical analysis

All analyses were performed using Stata version 14.2 (StataCorp LP, College Station, TX). Categorical variables are presented as percentages, and continuous variables are presented as means with standard deviations. Chi-squared tests were used to compare categorical variables, and two-tailed t tests were used for continuous variables. For regression analyses, multivariable logistic regressions were used for binary outcomes. Similarly, multivariable linear regressions were used for continuous outcomes.

### 2.6. Ethics statement

The protocol and study design were approved by the Ethics and Scientific Review Committee at AMREF Health Africa (Proposal number: AMREF ESRC P708-2019). Informed consent was obtained from all participants.

## 3. Results

A total of 234 CHWs across four cohorts in: North, East, South, and Central Kamagambo were included in the study (Table 1). South had the youngest cohort, with an average age of 30 at baseline and 41 at endline, the rest of the cohorts ranged from 41 to 45. The baseline survey included 81% female CHWs and the endline included 78%. The proportion of women ranged from 77 to 91% across all groups. The average years of experience caring for pregnant women ranged from 5.4 years in East to 9.7 years in North.

**Table 1:** CHW demographics.

	North Kamagambo		East Kamagambo		South Kamagambo		Central Kamagambo	
Variables	Baseline	Endline	Baseline	Endline	Baseline	Endline	Baseline	Endline
	*n* = 62	*n* = 63	*n* = 69	*n* = 78	*n* = 46	*n* = 52	*n* = 23	*n* = 38
Age in years								
Mean (Std. dev.)	41.87 (10.25)	42.95 (9.32)	40.42 (9.02)	41.97 (8.94)	30.22 (15.67)	40.96 (8.59)	44.87 (12.46)	44.84 (9.56)
Gender								
Female	50 (80.6%)	51 (80.95%)	53 (76.8%)	59 (75.64%)	38 (82.6%)	44 (84.62)	21(91.3%)	28 (73.68%)
Male	12 (19.4%)	12 (19.05%)	16 (23.2%)	19 (24.36%)	8 (17.4%)	8 (15.38%)	2 (8.7%)	10 (26.32%)
Education level								
Class 8 or less	32 (51.61%)	31 (49.20%)	24 (34.78%)	29 (37.18%)	18 (39.13%)	19 (36.54%)	10 (43.49%)	8 (21.05%)
Class 9 or higher	30 (48.39%)	32 (50.8%)	45 (65.22%)	49 (62.82%)	28 (60.87%)	33 (63.46%)	13 (56.52%)	30 (78.95%)
Literacy								
Passed test	32 (51.61%)	33 (52.38%)	51 (73.91%)	57 (73.08%)	32 (69.56%)	33 (63.46%)	11 (47.83%)	20 (52.63%)
Did not pass	30 (48.39%)	30 (47.62%)	18 (26.08%)	21 (26.92%)	14 (30.44%)	19 (36.54%)	12 (52.17%)	18 (47.37%)
Years caring for pregnant women								
Mean (Std. dev.)	9.71 (7.45)	9 (3.50)	5.40 (3.997)	7.04 (5.69)	8.17 (6.34)	9.08 (6.07)	7.69(6.2699)	7.05(5.42)

A total of 234 distinct CHWs were interviewed across the baseline and endline.

Education level varied by cohort (Table 1). At baseline and endline North had the lowest percentage of CHWs with a Class 9 education or higher (48, 51%). At baseline East had the highest percentage of educated CHWs (65%), while Central had the highest percentage at endline (79%).

We also conducted a literacy test which consisted of 7 questions, one would need to correctly score 6 out of the 7 questions (86%) to pass (Table 1). Across the CHWs surveyed at baseline, 63% passed the literacy test. Across the CHWs included at both baseline and endline, East had the highest literacy rate at (74, 73%) while Central had the lowest (48, 53%), with North performing only slightly higher (52, 52%).

At baseline, all CHWs in North and East had been trained through the intervention, while CHWs in South and Central had not (Table 2). At endline, South CHWs were newly trained and Central CHWs remained untrained by the intervention. At baseline 96–100% of CHWs in North, East, and Central reported being trained in obstetric and early infant danger signs. In comparison, only 70% of CHWs in South reported receiving training on obstetrics and 72% on early infancy. By the endline, these numbers in South, which had received the intervention between baseline and endline, increased to 96% for obstetrics and 95% for early infancy. In Central, which did not receive the intervention, the number of CHWs reporting that they had been trained in these topics decreased to 77% for obstetrics and 79% for early infancy. The training rates for North and East remained consistent. The average time since the last training on obstetrics and early infancy ranged from ~5 months in East to ~18.5 months in Central at baseline and ~ 7 months in East to ~18.5 in Central at endline.

**Table 2:** CHW training.

	North Kamagambo		East Kamagambo		South Kamagambo		Central Kamagambo	
Variables	Baseline	Endline	Baseline	Endline	Baseline	Endline	Baseline	Endline
	*n* = 62	*n* = 63	*n* = 69	*n* = 78	*n* = 46	*n* = 52	*n* = 23	*n* = 38
Trained through intervention								
Yes	62 (100%)	63(100%)	69 (100%)	78(100%)	0 (0%)	52 (100%)	0 (0%)	0 (0%)
No	0 (0%)	0 (0%)	0 (0%)	0 (0%)	46 (100%)	0 (0%)	23 (100%)	38 (100%)
Ever trained in obstetric danger signs								
Yes	62 (100%)	63 (100%)	67(97.1%)	75 (96.15%)	32 (69.6%)	51(98.08%)	22 (95.6%)	29 (76.32%)
No	0 (0%)	0 (0%)	2 (2.9%)	3 (3.85%)	11 (23.9%)	1 (1.92%)	1(4.4%)	9 (23.68%)
Missing					3 (6.5%)			
Ever trained in neonatal danger signs								
Yes	62(100%)	63 (100%)	68 (98.6%)	75 (96.15%)	33 (71.7%)	51 (98.08%)	22 (95.6%)	29 (76.32%)
No	0 (0%)	0 (0%)	1 (1.4%)	3 (3.85%)	10 (21.7%)	1 (1.92%)	1(4.4%)	9 (23.68%)
Missing					3 (6.5%)			
Months since last training on obstetric danger signs								
Mean (Std. dev.)	8.85 (6.70)	9.25 (8.226)	5.03 (4.25)	6.78 (9.326)	9.3 (17.04)	5.37 (2.95)	18.39 (15.59)	25.31 (11.72)
Months since last training on neonatal danger signs								
Mean (Std. dev.)	7.64 (5.56)	9.19 (8.30)	4.87 (4.24)	6.68 (9.34)	6.24 (9.91)	5.37 (2.95)	18.8 (15.82)	25.31 (11.72)

A total of 234 distinct CHWs were interviewed across the baseline and endline.

We assessed CHWs’ knowledge across pregnancy, childbirth, infancy, and the postpartum period, considering a CHW knowledgeable if they were able to correctly identify three or more danger signs (Table 3). Across all cohorts, CHWs were more knowledgeable about danger signs associated with pregnancy and were least knowledgeable about danger signs during childbirth. At baseline, North -the longest-standing intervention site -scored the highest on all 4 knowledge domains with an average 77% of CHWs considered knowledgeable, compared to the other cohorts which ranged from 59–72%. At endline, there were significant increases in knowledge scores across all three cohorts in the intervention: North, East, and South. East, which received the intervention shortly before the baseline, saw its proportion of CHWs passing increase from 62 to 92% with statistically significant change across all 4 knowledge domains. (*p* = 0.0001, *p* = 0.0004, *p* = 0.0004, p = <0.0001). South, which joined the intervention between the baseline and endline, saw an increase from 59 to 82% with significant change across all domains (*p* = 0.0003, *p* = 0.0124, *p* = 0.0116, *p* = 0.0253). CHWs in Central, which had not received the intervention, only saw an increase in knowledge of pregnancy from 78.3 to 81.58% (*p* = 0.0253), and actually saw decreases across the other three domains, though those changes were not statistically significant.

**Table 3:** CHW Knowledge.

	North Kamagambo			East Kamagambo		
% knowledge per category	Baseline	Endline	value of *p*	Baseline	Endline	value of *p*
	*n* = 62	*n* = 63		*n* = 69	*n* = 78	
Pregnancy	52 (83.9%)	63 (100%)	0.0016	53 (76.8%)	77 (98.72%)	0.0001
Childbirth	42 (67.7%)	49 (77.78%)	0.4913	36 (52.2%)	67 (85.9%)	0.0004
Postpartum	45 (72.6%)	57 (90.48%)	0.029	42 (60.9%)	67 (85.9%)	0.0004
Early infancy	52 (83.9%)	55 (87.30%)	0.2059	40 (58%)	75 (96.15%)	0
Average	77.25%	88.75%		62.00%	91.75%	
Mean Score (Std. dev)						
Pregnancy	4.61 (2.15)	5.29 (1.61)		3.41(1.57)	5.24 (1.43)	
Childbirth	3.47 (1.65)	3.48(1.25)		2.67 (1.44)	4.14(1.53)	
Postpartum	3.48 (1.52)	4.11(1.38)		3.03(1.57)	4.41(1.65)	
Early infancy	3.76 (1.25)	3.65(1.3)		2.85 (1.1791)	4.18(1.03)	
	South Kamagambo			Central Kamagambo		
% knowledge per category	Baseline	Endline	value of *p*	Baseline	Endline	value of *p*
	*n* = 46	*n* = 52		*n* = 23	*n* = 38	
Pregnancy	33 (71.7%)	49 (94.23%)	0.0003	18 (78.3%)	31 (81.58%)	0.0253
Childbirth	21 (45.6%)	38 (73.08%)	0.0124	15 (65.2%)	17 (44.74%)	1.000
Postpartum	27 (58.7%)	41 (78.85%)	0.0116	14 (60.9%)	24 (63.16%)	0.0956
Early infancy	26 (56.5%)	42 (80.77%)	0.0253	19 (82.6%)	21 (55.26%)	0.3173
Average	58.50%	81.75%		71.75%	61.25%	
Mean Score (Std. dev)						
Pregnancy	3.46(1.6)	4.46 (1.59)		4.22 (2.09)	4.18 (1.8)	
Childbirth	2.39 (1.31)	3.11(1.28)		2.96 (1.52)	2.95(2.01)	
Postpartum	2.739 (1.32)	3.27(1.05)		3.30 (1.64)	3.5(1.93)	
Early infancy	2.69 (1.19)	3.21(1.07)		3.22 (1.24)	3.13(1.68)	

^*^value of *p* calculated using McNemar’s chi2.

^**^A total of 234 distinct CHWs were interviewed across the baseline and endline.

Next, we used logistic regression to assess education, literacy, experience, training, and gender as potential predictors of CHW knowledge (Table 4). We found recency of training to be associated with increased knowledge of danger signs in pregnancy (OR = 1.06; CI = 1.002, 1.124; *p* = 0.044) and early infancy (OR = 1.05; CI = 0.996, 1.099; *p* = 0.07). Experience caring for pregnant women was also associated with increased knowledge of danger signs in pregnancy (OR = 1.08; CI = 0.989, 1.187; *p* = 0.086) and early infancy (OR = 1.08; CI = 0.987, 1.099; *p* = 0.094).

**Table 4:** Logistic regressions, knowledge.

	Pregnancy				Neonatal			
	Odds ratio	95% Confidenc interval		value of *p*	Odds ratio	95% Confidenc interval		value of *p*
Trained in Intervention	1.838	0.866	3.901	0.113	1.79	0.853	3.758	0.124
Educated (> class 8)	1.232	0.595	2.552	0.574	1.103	0.55	2.214	0.782
Literate	1.502	0.715	3.154	0.283	0.413	0.193	0.886	0.023*
Female	0.951	0.383	2.363	0.914	0.815	0.337	1.97	0.649
Recency of training (months)	1.061	1.002	1.124	0.044^*^	1.046	0.996	1.099	0.07*
Experience (years)	1.083	0.989	1.187	0.086^*^	1.078	0.987	1.178	0.094^*^
Constant	0.641	0.163	2.522	0.525	1.279	0.323	5.069	0.727
	Childbirth				Postpartum			
	Odds ratio	95% Confidenc interval		value of *p*	Odds ratio	95% Confidenc interval		value of *p*
Trained in Intervention	1.535	0.814	2.895	0.185	1.316	0.678	2.552	0.417
Educated (> class 8)	1.117	0.605	2.06	0.724	1.882	0.997	3.553	0.051*
Literate	0.734	0.39	1.38	0.337	0.665	0.34	1.301	0.234
Female	1.008	0.48	2.118	0.982	1.232	0.572	2.654	0.595
Recency of training (months)	1.001	0.976	1.028	0.921	0.985	0.959	1.013	0.299
Experience (years)	1.045	0.981	1.112	0.173	1.048	0.979	1.123	0.177
Constant	0.765	0.248	2.364	0.642	0.927	0.284	3.019	0.899

^*^means statistically significant.

We then used multiple linear regression to assess predictors of CHW performance (Table 5). CHW participation in the intervention training was associated with a 15% increase in the likelihood of a child being fully immunized at 12 months (Coef = 15.47; CI = 11.41, 19.53; *p* = 0) and a 14% increase in the likelihood of a mother completing four or more antenatal care visits (Coef = 13.68; CI = 3.84, 23.53; *p* = 0.007).

**Table 5:** Linear regressions, performance.

	Immunization				ANC			
	Coef.	95% Confidence interval		value of *p*	Coef.	95% Confidence interval		value of *p*
Trained in Intervention	15.471	11.415	19.526	0*	13.683	3.837	23.528	0.007*
Educated (> class 8)	0.736	−2.638	4.11	0.667	−3.975	−12.504	4.554	0.358
Literate	3.518	−0.478	7.514	0.084^*^	4.875	−4.603	14.354	0.311
Female	0.904	−2.908	4.716	0.64	8.177	−4.11	20.464	0.19
Recency of training (months)	0.11	−0.084	0.303	0.264	−0.259	−1.028	0.509	0.506
Experience (years)	0.037	−0.384	0.458	0.863	−0.332	−1.167	0.503	0.433
Constant	77.991	70.004	85.977	0	73.503	54.676	92.329	0

^*^means statistically significant.

Interestingly, there was minimal collinearity between education and literacy, so we included both in the regression models. Having completed schooling beyond Class 8 was associated with increased knowledge of postpartum danger signs (OR = 1.88, CI = 0.997, 3.552; *p* = 0.051) however, education was not predictive of either of the performance measures. Literacy was actually negatively associated with knowledge of neonatal danger signs (OR = 0.41, CI = 0.193, 0.886, *p* = 0.023) however it was positively associated with immunization rates (Coef = 3.52, CI = −0.478, 7.514, *p* = 0.084). There was no association between gender and knowledge or performance.

## 4. Discussion

### 4.1. Knowledge

All three cohorts in the intervention saw increases in knowledge scores from baseline to endline. South, which started the intervention between baseline and endline, saw a significant increase. However, so did East, which started 1 year before the baseline and North, which had started the full intervention more than 2 years prior. These knowledge gains were not seen in the Central, which had not received the intervention, and actually saw a decrease in knowledge over the study period. This provides a helpful comparison and suggests the intervention contributed to increased knowledge among CHWs. Additionally, the literature finds that CHW knowledge often drops after initial training (36, 37). The fact that knowledge for CHWs in the North and East cohorts actually increases overtime may be explained by the connection between recency of training and knowledge found in the regression analysis (Table 4). Further research is required to enumerate the connection between frequency of refresher training and the retention of knowledge among CHWs (31).

### 4.2. Training

Whether the CHW had been trained through the intervention was a significant predictor of immunization rates and antenatal care completion rates amongst CHW clients. We would expect the intervention to have less impact on CHW performance as compared to CHW knowledge since non-intervention factors may have influenced ANC completion and immunization rates during the study period. Additionally, it is important to note that nearly all CHWs trained by the intervention also received the other program components including consistent payment and frequent supervision. Therefore, it is not possible to associate these results to training independent of these other elements. Rather, training could be viewed as a proxy for CHW professionalization, which includes supervision and payment. Our results are consistent with the wider literature which finds payment, frequent supervision, and continuous training to be associated with increased CHW performance (12). Other research shows training of TBAs to be positively associated with increases in knowledge and antenatal care outcomes (38, 39). We would like to see future studies explore the impact of this intervention on TBAs who have been incorporated into CHW cadres and compare competence of these transformed TBAs with other CHWs.

### 4.3. Experience

Year of caring for pregnant women is a predictor of increased knowledge of danger signs in pregnancy and early infancy, but was not associated with CHW performance outcomes. Most of the research in this area focuses on experience as a CHW specifically, and the results are mixed. A study in Kenya finds positive association between experience as a CHW and use of job aids, client satisfaction, and client enablement (40), conversely a study in Uganda found that CHWs who had serve for more than 6 years had worse client outcomes than CHWs who had served for less (31). Since our study looked at *any* experience caring for pregnant women it also may be capturing the experience of CHWs who previously acted at TBAs. Further, we call for more research on the link between experience and CHW performance.

### 4.4. Education and literacy

Our results on the connection between education and literacy and CHW knowledge and performance are mixed. Education was positively associated with one of the four knowledge domains, while literacy was negatively associated with another. Literacy was associated with performance on immunization rates but not antenatal care completion, and the effect size of literacy and immunizations rates (Coef = 3.52, CI = −0.48, 7.51, *p* = 0.084) are less strong than the effect size of training on the same variable (Coef = 15.47; CI = 11.41, 19.53; *p* = 0). Finally, formal education was not associated with either of the performance measures.

This incertitude matches the wider literature (31). A 2016 study of notably low performing CHWs in Northern Uganda found education to be positively correlated with performance (24). Similar results were found in Kenya (25) and Madagascar (26). However, studies in Siaya Kenya, Central Uganda, and South Sudan did not find education to be predictive of good performance (27, 28, 30). Neither did a study of the Living Goods CHW selection process in Kenya which included written tests and one-to-one interviews, but could not find an association between these screens and performance (29). Interestingly, a cross-sectional study in Nigeria which included TBAs in CHW cohorts, argued that properly trained CHWs with lower education levels can be as knowledgeable as CHWs with high education levels (41).

Together with the results of this study we conclude that education and literacy are not reliable factors associated with CHW competency and deficiencies in education might be overcome by other design factors such as frequent training and supervision. Since many CHWs in Kenya and globally are illiterate or semi-literate, and education and literacy requirements are more likely to bias against women, this is an important topic for further inquiry.

### 4.5. Gender

Across our measures of knowledge and performance we found no significant association with gender. However, studies which used different indicators of performance did find associations. For example, a 2012 study in Busia, Kenya found that male CHWs were more likely to keep satisfactory records, while female CHWs were more likely to counsel clients and influence behavior change (40) and a 2012 study in Uganda found that female CHWs were less likely to lose clients to follow up (42). We recommend further inquiry, especially including less traditional measures of competency like empathy and trust.

### 4.6. Study limitations

Limitations include the fact that all four CHW cohorts are geographically adjacent to each other. So even though South CHWs did not receive the intervention at the baseline and Central CHWs did not receive the intervention prior to either survey, there may have been spillover effects. This is especially true as the study period took place in the midst of COVID-19 and both Lwala Community Alliance and the Ministry of Health provided additional training and support, including personal protective equipment, to most CHWs in the study, including many in Central.

Additionally, the study did not randomly select the intervention areas. Instead the CHWs received the intervention according to programmatic and demographic considerations led by the Ministry of Health. Another limitation is that the study does not include other intervention elements that may have influenced knowledge and performance, including payment and supervision, instead training acts as a proxy for all of these elements as they were added as a package. Finally, while 18% of CHWs in study were former TBAs we did not have sufficient numbers across the intervention and non-intervention cohorts in both baseline and endline to include experience as a TBA in our analysis.

## 5. Conclusion

Our study finds that the intervention’s training was significantly associated with performance of CHWs. Further inquiry is recommended to investigate the influence of other program components, including payment, supervision, and refresher training. We also found recency of training and experience caring for pregnant women to be predictive of knowledge of pregnancy and postpartum danger signs. In comparison, the link between education and literacy and CHW performance and knowledge were inconsistent. Further, we postulate that the deficiencies presented by lower education or literacy levels may be recovered by robust training and supervision. Policy makers and practitioners should reconsider the use of education and literacy requirements in the selection process of CHWs, especially if these design choices are intended to influence knowledge or performance. Instead, we encourage selection to focus on community acceptability and experience caregiving and for programs to use competency-based assessments and accreditation following CHW training. We encourage further research into the selection criteria for CHWs and intervention design choices that may influence CHW performance. We think this inquiry would be particularly valuable in the context of a non-NGO setting in which government it working to professionalize CHWs through payment, supportive supervision and training.

## Data availability statement

The raw data supporting the conclusions of this article will be made available by the authors, without undue reservation.

## Ethics statement

The studies involving human participants were reviewed and approved by Ethics and Scientific Review Committee at AMREF Health Africa (Proposal number: AMREF ESRC P708-2019). The patients/participants provided their written informed consent to participate in this study.

## Author contributions

JS and AR developed the original study protocol. VO, LG, SO, and JS contributed to the refinement of the study protocol. LG, AR, JS, and JW prepared the initial draft of this manuscript. LG, SM, SO, and AR provided input into initial and final refinements of the full manuscript. All authors contributed to the article and approved the submitted version.

## Funding

Costs for this study were paid for by Lwala Community Alliance’s unrestricted funds and were not financed by any other donor source.

## Conflict of interest

The authors declare that the research was conducted in the absence of any commercial or financial relationships that could be construed as a potential conflict of interest.

## Publisher’s note

All claims expressed in this article are solely those of the authors and do not necessarily represent those of their affiliated organizations, or those of the publisher, the editors and the reviewers. Any product that may be evaluated in this article, or claim that may be made by its manufacturer, is not guaranteed or endorsed by the publisher.

## Abbreviations

CHW: Community Health Worker
TBA: Traditional Birth Attendant
